# Ultrathin water layers on mannosylated gold nanoparticles

**DOI:** 10.3762/bjnano.16.151

**Published:** 2025-12-04

**Authors:** Maiara A Iriarte Alonso, Jorge H Melillo, Silvina Cerveny, Yujin Tong, Alexander M Bittner

**Affiliations:** 1 CIC nanoGUNE (BRTA), Donostia, Spain; 2 Tecnipesa Identificacion SL, Parque Empresarial Zuatzu, Donostia, Spain; 3 Centro de Fisica de Materiales (CFM-MPC, CSIC-UPV/EHU) Donostia, Spain; 4 Donostia International Physics Center (DIPC), Donostia, Spain; 5 Fritz-Haber-Institut der Max-Planck-Gesellschaft, Berlin, Germany; 6 Fakultät für Physik, Universität Duisburg, Germany; 7 Ikerbasque Basque Foundation for Science, Bilbao, Spain

**Keywords:** AFM, humidity, hydrophilicity, hydrophobicity, nanoparticles, sum frequency generation spectroscopy, viruses, water, wetting

## Abstract

We investigated the effect of air humidity on two gold nanoparticle systems, one functionalized with an oligo(ethylene glycol) ligand, and one functionalized with a mixture of the same with a dimannoside ligand. The dimannoside ligand was chosen to mimic the surface chemistry of viral spike proteins. We characterized the particles by electron microscopy, dynamic light scattering, and infrared spectroscopy. We probed particles adsorbed on hydrophilic and hydrophobic surfaces with atomic force microscopy (AFM) and vibrational sum frequency generation (VSFG) spectroscopy, both operated under variable air humidity. For AFM, we additionally tested hydrophilic and hydrophobic tips. While VSFG indicated preferential hydration of the dimannoside and proved conformational changes in the organic ligands, AFM provided sub-nanometer changes in particle topography due to water adsorption. In general, the dimannoside nanoparticles condense ultrathin water layers upon humidity increase. In contrast, we found that the water adsorption on the oligo(ethylene glycol) particles depends little on humidity. Our insights into structural changes on glyconanoparticles and the hydration properties of glycosylated particles are of application value for biosensors and help model the transmission of airborne viruses, such as influenza.

## Introduction

Gold nanoparticles (AuNPs) have been a staple in biomedical and biophysical research [1–2] for almost a century [3]. They are investigated, for example, regarding drug delivery [4], but they are also parts of actual products, for example, of sensors [5]. All this is based on the ease of synthesis, chemical stability, size tuneability, and unique optical properties [6]. The extreme dependence of the properties on particle size and shape has been demonstrated for particle sizes in the 1–100 nm range and on biological interfaces [7]. Limited biocompatibility and high tendency to aggregate in solution inspired new mechanisms of particle biofunctionalization with proteins, lipids, or carbohydrates. Coupling carbohydrates to AuNPs provides particle stability and biocompatibility and allows for studying carbohydrate-mediated interactions and designing novel carbohydrate-based antiviral agents [8–9]. From a molecular point of view, glyconanoparticles are water-soluble gold nanoclusters with a three-dimensional carbohydrate display, which defines their biological function [10], a small globular core, and chemically well-defined composition. Proof-of-concept studies have demonstrated the vast potential of glyconanoparticles for glyconanotechnology in solution. However, potential changes of “glycoclusters” [2] in dry or humid environments are not well known, although there are many examples, for example, sensors that use antibodies (glycosylated proteins) linked to AuNPs, such as the now very established SARS-CoV-2 antigen tests [5]. While practical questions about storage conditions and lifetimes call for tests in a realistic environment, the scientific bases are assumptions and analogies to chemically similar systems, rather than data.

Several authors have synthesized and investigated (di)mannoside-coated AuNPs. While there are multiple applications [4], such NPs can also be seen as very crude models of viral spikes, which are crucial for virus “survival” during transmission [11–12]. For many, especially mammalian, viruses, transmission occurs in aqueous environments (e.g., Dengue [13] and Ebola [14]), and air humidity does not play any direct role. In contrast, humidity strongly affects the transmission of influenza [11–12,15] or SARS-CoV-2 [14] via aerosols. These viruses are enveloped (by glycosylated lipid bilayers) and display very large multimers of nanoscale glycoproteins (i.e., spikes), which control virus attachment and fusion to the host cells [16]. Glycosylation, often with mannosides [17], is essential to infection. Complete dryness is certainly detrimental (lipid bilayers ultimately collapse). Still, how such virus surfaces are preserved under low-humidity conditions, as is typical for airborne transmission in Northern Hemisphere winters, is unknown [18]. We believe that a glycosylated AuNP can provide a simplified model of a viral spike, whenever the virus is very densely coated, for example, influenza by hemagglutinin [18–19]. Hence, our idea is not to emulate complete virions; rather, one NP is emulating one single spike protein. Although the density of the NPs is not very high and the shape cannot be identical (hemagglutinin is roughly a triangular 7 nm prism of 15 nm length), the size is in the typical NP range, and a dense coating with oligomannoside should mimic surface physics. Accordingly, we note that the adsorption of AuNPs on surfaces would mimic the survival of adsorbed viruses, which can either be transmitted mechanically or again become airborne. In any case, the role of air humidity for adsorbed viruses is poorly documented, and its influence on transmission physics is not known.

**Figure 1:**
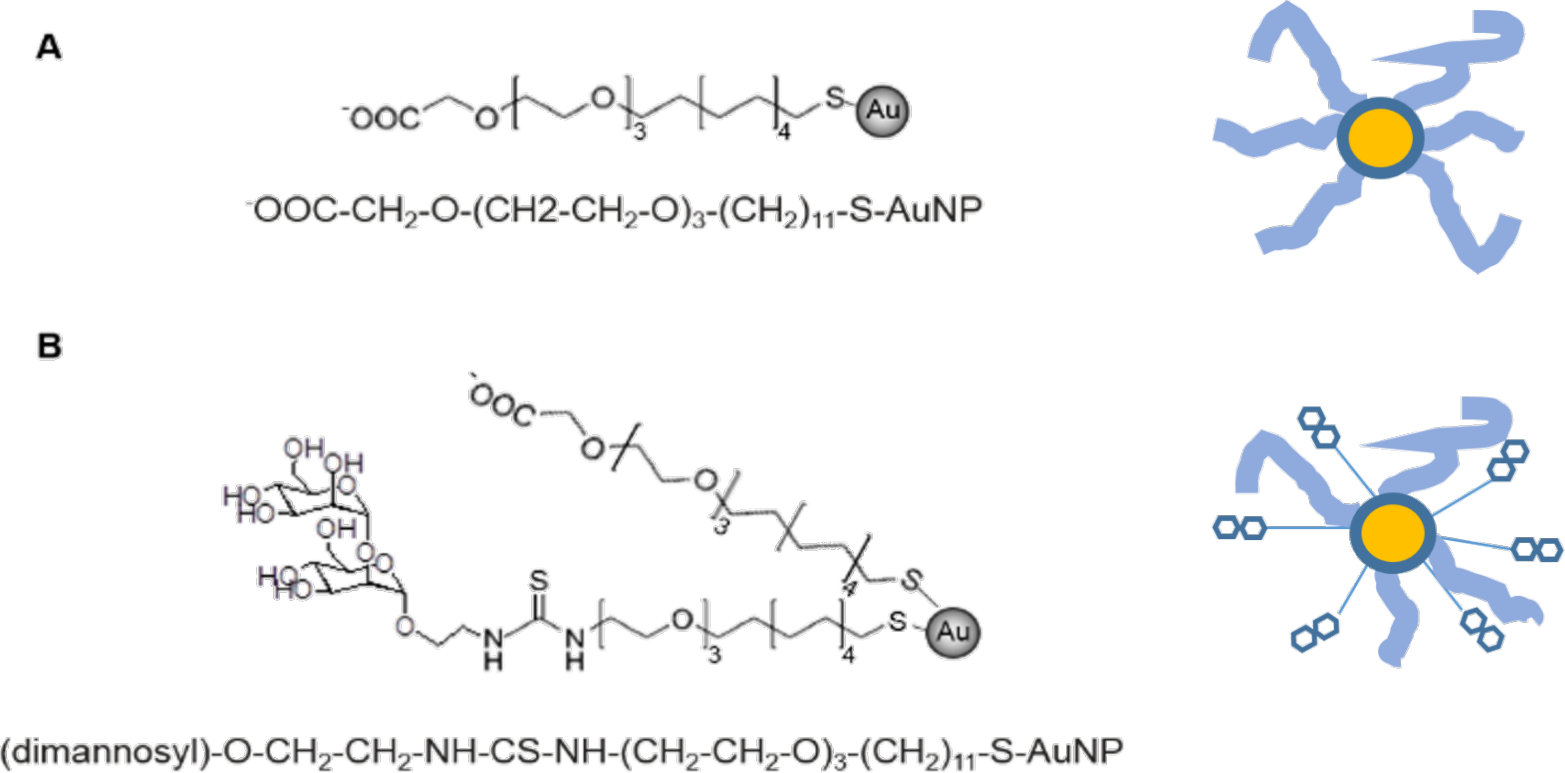
Structures of gold nanoparticles used in this work. (A) PEG AuNPs were obtained in the presence of carboxyl PEG thiol. (B) Dimanno-AuNPs were covered with 50% of dimannoside and 50% of carboxyl PEG thiol. Note that, in (A) and (B), the linear structure of the particles is displayed below the structural scheme. In (B), only the dimannoside is displayed. The yellow circles represent the gold core, the light blue lines the PEG ligand, and the thin lines the PEGylated ligand terminated by dimannose residues (two hexagons).

We chose as models dimannoside gold nanoparticles (dimanno-AuNPs) [6] linked to a thiourea PEG thiol chain; the particles also feature COOH-terminated PEG chains (Figure 1). We used a typical standard PEG coating on AuNPs, again with COOH termini (PEG AuNPs) for comparison. Here, and in the following, we use the established term “polyethylene glycol” (PEG) to designate our relatively short oligo(ethylene glycol) chains.

The particles were first characterized by dynamic light scattering (DLS) and zeta potential (ZP) measurements in solution, and by scanning electron microscopy (SEM) and scanning transmission electron microscopy (STEM) in vacuum. Samples were adsorbed on flat inorganic surfaces, usually modified with organic layers, and probed by Fourier-transform infrared spectroscopy (FTIR), vibrational sum frequency generation (VSFG), and atomic force microscopy (AFM). For VSFG and AFM, we systematically varied the relative air humidity (RH).

DLS and ZP yield particle size and stability in solution, in terms of hydrodynamic diameter and NP surface charge, respectively. Spectroscopy techniques were used to analyze the chemical composition of the organic ligands locally. We used FTIR for the molecular fingerprint infrared region to find the characteristic peaks of the organic layers. In contrast, VSFG was applied to obtain interface-sensitive information on CH and OH bonds at the AuNP/air interface, under hydration and dehydration. We also used a deuterated water (D_2_O) atmosphere to distinguish the mannosyl hydroxy groups from adsorbed and absorbed water. Absorption is not possible inside the NPs, but inside the ligand sphere, especially between dimannoside groups, but possibly also between PEG chains. The resulting expansion of the ligands cannot be distinguished from adsorption of water, that is, when water molecules are selectively placed on the ligands. In either case the NPs will expand. We will, however, demonstrate the role of the ligand conformation.

We obtained detailed spatial characterization by SEM, to measure size and shape, and to detect aggregation upon adsorption on surfaces of different hydrophilicity (see Supporting Information File 1, Section S1). We used heavy metal staining in STEM on the nanometer scale, to distinguish the organic ligand shell from the gold core. The main method, however, was “noncontact” (AC mode) AFM. Its advantage lies in obtaining a very detailed surface topography through height images. This also includes adsorbed water layers on the sample, which differ from the adsorption on the surface, such that height variations taking place only on the sample are correlated with air humidity [20]. These ideas are inspired by previous works of the group [18,20–21].

Ultrathin water layers are extremely delicate, and there is a risk of over-interpreting height information. Indeed, experiments with soft matter in ambient humidity usually create a thin film of water covering the tip and the sample. A capillary water neck is formed in hydrophilic systems when the AFM tip comes close to the sample surface [22]. In unfavorable cases, this can result in apparent heights up to four times larger than the actual values [23]. Experimentally, this issue can be reversed by different approaches. First, the height measurements can be achieved at set point ranges and working distances where only attractive regimes in the amplitude–distance curves are accessed. Second, tips with different hydrophilicity complement topographic information with accurate height determination. Third, the same is true for surfaces; self-assembled monolayers (SAMs) of silanes are well suited to assess the water layer contribution in AFM measurements [24]. They form stable and well-defined organic layers on oxides, for example, on oxidized silicon wafers or glass, where surface charge and hydrophilicity are controlled by selecting the appropriate end groups. We combined all the methods mentioned to carry out AFM at variable RH levels in our AuNP systems. Specific care was taken during height measurements, as demonstrated by amplitude–distance curves and statistical analysis (*t*-tests). We added experiments with deuterated water analogous to the mentioned VSFG tests.

## Results

The dimanno-AuNPs consist of functionalized AuNPs with a mixture of dimannoside and PEGylated ligands. The PEG AuNPs were functionalized only with the PEGylated component; they can be seen as a precursor, and we used them as negative controls.

### Particle size and shape

**Figure 2:**
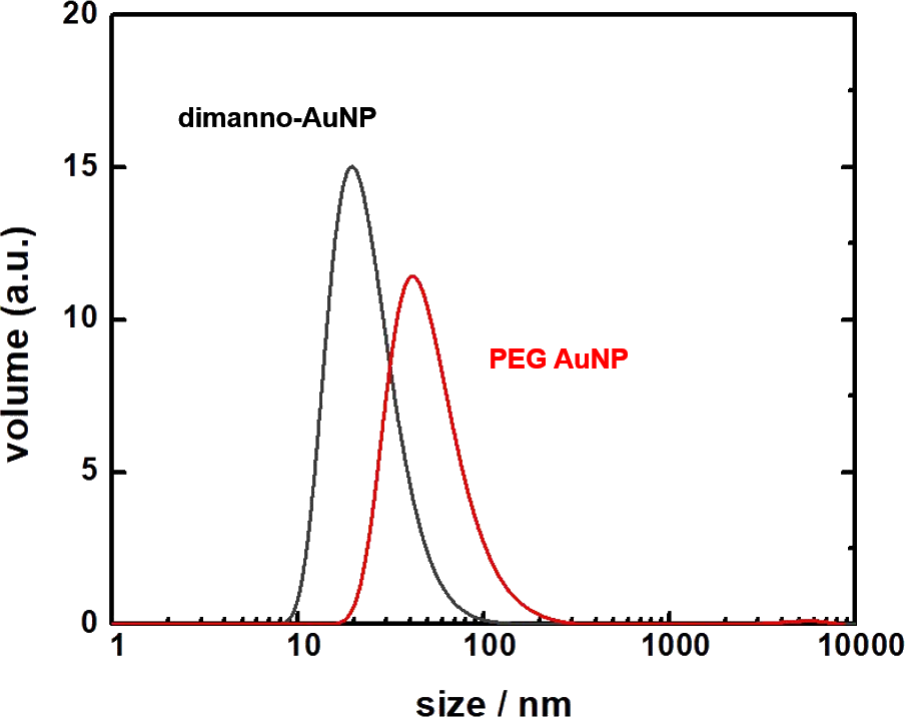
Distribution of hydrodynamic diameters of dimanno-AuNP (black) and PEG AuNP (red) solutions, obtained by DLS.

Particle size and stability in an aqueous solution were characterized by DLS and ZP measurements, while size and morphology under completely dry conditions were measured by electron microscopy. DLS yields a hydrodynamic diameter of the PEG AuNPs of 67.7 ± 9.4 nm and a polydispersity index (PDI) of 0.35. However, the size distribution (Figure 2) reaches a maximum at 41.1 ± 4.2 nm. In contrast, the size average of the dimanno-AuNPs is 30.4 ± 1.1 nm (PDI = 0.29) with a peak at 16.8 ± 0.9 nm. This indicates that the dimannoside coating results in reduced nanoparticle aggregation. Hence, a small number of multimers of the NPs is present in the solution (i.e., stronger particle association). DLS is inherently sensitive to the hydrodynamic radius, and therefore to aggregation, even when the core sizes are similar. A more hydrated surface is typically associated with higher colloidal stability and lower aggregation, which is consistent with the smaller hydrodynamic size observed by DLS for the dimanno-AuNPs.

In both cases, the NP surface charge in water at pH ~7 was approx. −20 mV. The isoelectric point of carboxylate PEG-capped particles is ~2.5; hence, around pH 7, they should exhibit a negative ZP. This is also compatible with a low carboxylate content, as citrate-capped AuNPs (with a higher concentration of carboxylate) exhibit a lower ZP (approx. −45 mV) at the same pH [1].

**Figure 3:**
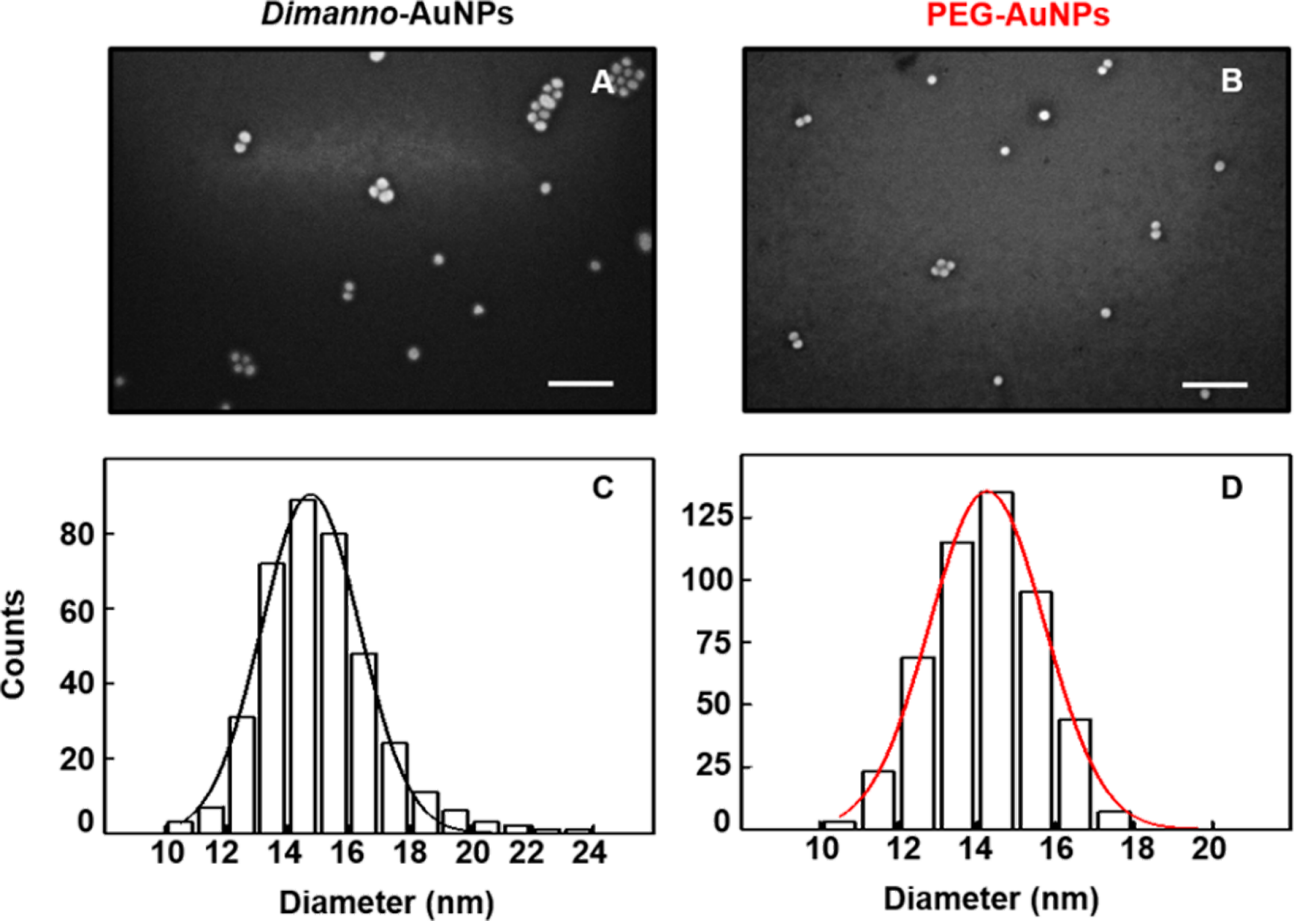
SEM images of dimanno-AuNPs and PEG AuNPs adsorbed to a hydrophilic surface. (A) Dimanno-AuNPs and (B) PEG AuNPs (scale bars: 100 nm). (C, D) Particle diameter histograms from (A) and (B), respectively.

To obtain a clearer view of the size distribution, we used SEM to evaluate particle sizes and morphologies in high vacuum (i.e., for completely dried samples). By adjusting a Gaussian fit to the histograms (Figure 3), the diameter of the particles adsorbed on 3-(ethoxydimethylsilyl)propylamine (APDMES) silicon was estimated. The dimanno-AuNPs exhibited a mean size of 14.8 ± 1.6 nm (Figure 3A,C), and the size of the PEG AuNPs was 14.3 ± 1.5 nm (Figure 3B,D).

**Figure 4:**
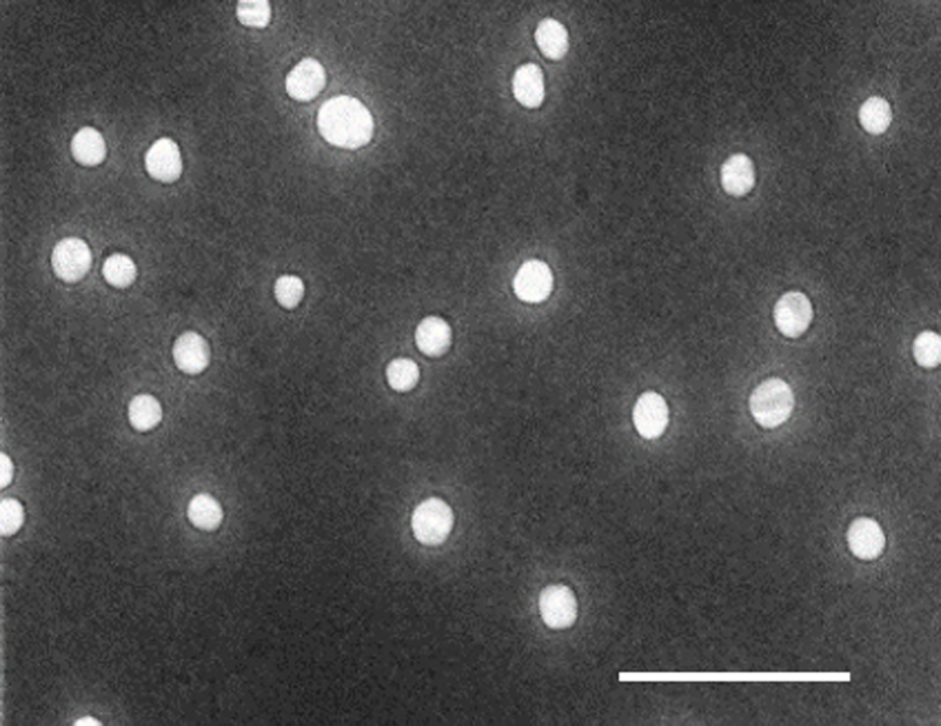
STEM image of negatively stained dimanno-AuNPs (scale bar 100 nm). The gold cores appear bright, the carbon grid background is dark, and the organic layer appears as grey halo.

The organic layers of PEG and dimannoside are transparent to electrons in SEM (only the gold cores were observed as bright features). We recorded STEM images of dimanno-AuNPs deposited on a carbon-coated TEM grid to visualize the layer. We employed uranyl as a stain, a soluble heavy-metal cation that attaches to the hydrophilic parts of the organic coating, providing good contrast [25]. The STEM images in dark-field mode (imaging scattered electrons) show the gold cores as bright structures, whereas the thin organic layers are darker. The carbon grid yields practically no scattering and appears essentially black, such that both the core and shell of the particles can be distinguished (Figure 4). The estimated organic layer thickness is 1.5 nm, as obtained by subtraction of the total radius of the negatively stained particles and the radius of the gold cores. Measuring center-to-center distances between adjacent gold cores and subtracting the core diameter indeed yields an independent estimate of the shell thickness. This analysis gives values consistent with the ~1.5 nm thickness extracted from single-particle measurements.

### Analysis of the chemical groups

The nature of the coating layer of PEG AuNPs and dimanno-AuNPs was characterized by FTIR (only in dry nitrogen) and VSFG at variable humidity. While the preparation involved a known stoichiometric ratio of the thiol mixture (50:50), our FTIR and VSFG data are primarily qualitative and semi-quantitative, serving to validate the presence of the desired chemical moieties. We discuss details of the FTIR spectra in Supporting Information File 1, Section S2, including the origin of the different bands. Here, we mention that the PEG AuNPs spectra exhibit only a trace of O–H stretching bands, in stark contrast to dimanno-AuNPs. It is well known that sugars are very difficult to dry completely, so related bands are easily found in many carbohydrates (see Supporting Information File 1, Section S2).

For the VSFG, the ssp-polarized spectra were obtained by adding a droplet of H_2_O or D_2_O on the dried samples with subsequent evaporation. The VSFG spectra focus only on the high-frequency region. The vibrational bands are superimposed on a very broad signal from bulk gold, quasi-constant after normalization (like FTIR). In contrast to FTIR, the bands stem only from vibrations at interfaces, that is, from the NP surface in contact with air. Additionally, highly symmetric vibrations are forbidden (with very low or zero intensities) [26].

The PEG signals correspond, as expected, quite well to the infrared spectrum, with a strong peak at 2880 cm^−1^. This feature points to a lack of local order, which is presumably based on disorder of the PEG chain (ordered alkyl chains, for example, yield no signal from CH_2_). Moreover, the peak is unusually broad, possibly due to the complex shape of the disordered PEG chains. However, this type of disorder refers to the conformation of the PEG chains only; altogether, the C–H stretching vibrations must have a preferred orientation with respect to the gold surface. In other words, the nanoparticle has a non-centrosymmetric environment, which can simply be the presence of the gold surface. The noise-like feature at ~3400 cm^−1^ is assigned to hydrogen-bonded water as features above 3600 cm^−1^ should indicate non-hydrogen-bonded water. This is proof that water is adsorbed on the PEG AuNPs. A quantification is not possible; however, there cannot be more than a few monolayers, otherwise the band would completely dominate the spectrum. This is also in agreement with the absence of the band in the FTIR spectra. Hence, the hydrophilic PEG chain binds water, but can easily be dried, while the traces of water remain. This is similar to a strongly bound water (mono)layer on biomolecules.

The dimanno-AuNPs, under the same conditions, exhibit a very different spectrum. The C–H stretching bands at 2850 and 2920 cm^−1^ (see also FTIR measurements) are “negative”, that is, the background VSFG signal is diminished. The frequency only slightly shifts from that of CH_2_ groups in alkyl and PEG chains. The dip-like features should be due to the interference effect between the resonant and the non-resonant signal. The different features of PEG-Au and dimanno-Au may be due either to the other dipole orientation of the CH group between the two samples [27], or to the charges of the molecules being opposite [28]. The latter seems unlikely since the isoelectric points of the two samples are similar.

**Figure 5:**
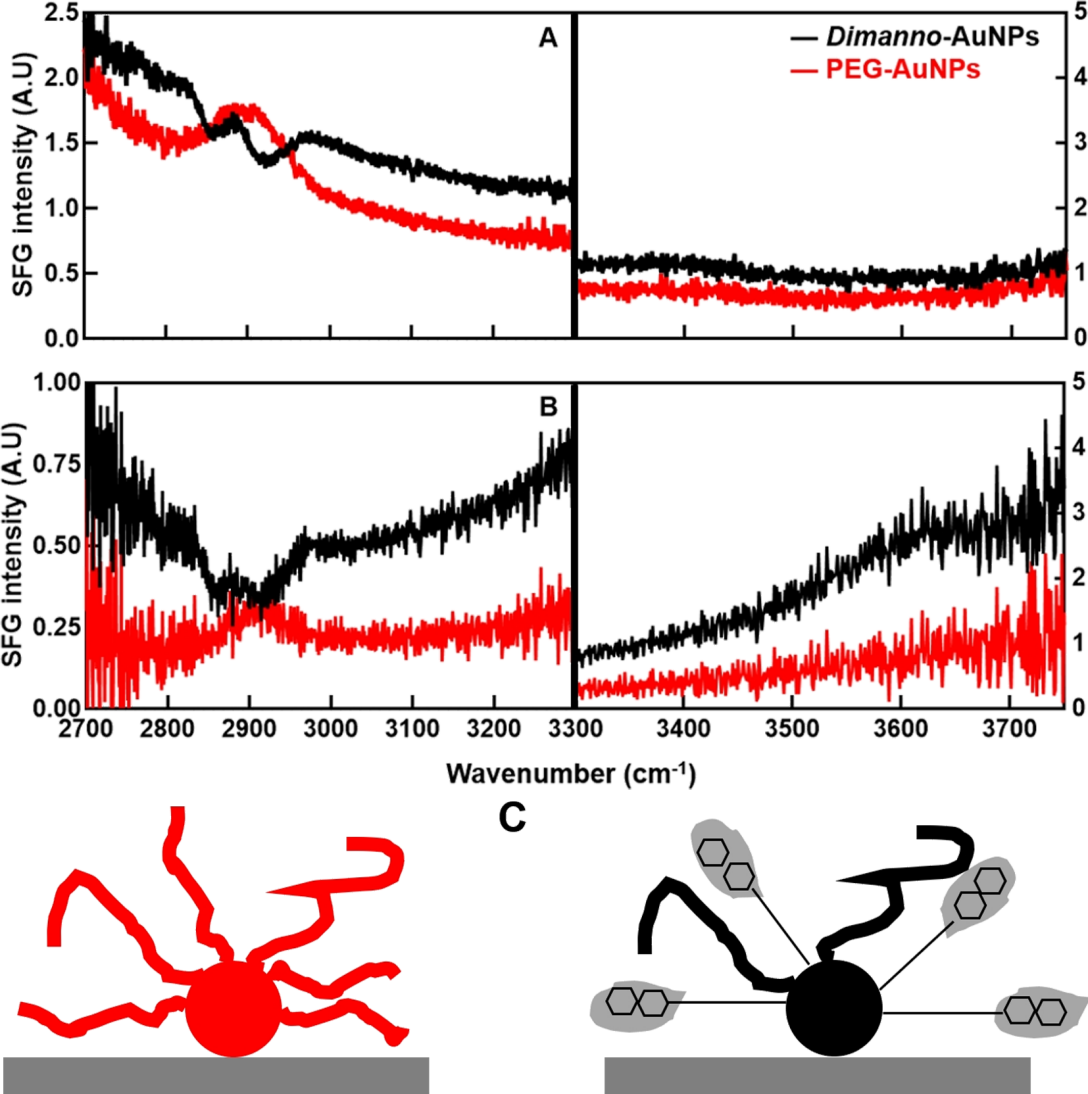
Normalized VSFG spectra in the CH region (<3300 cm^−1^) and the OH region (>3300 cm^−1^) of dimanno-AuNPs (black) and PEG AuNPs (red) on a gold surface for the ssp (s VSFG, s vis, p IR) polarization combination. (A) VSFG spectra in H_2_O. (B) VSFG spectra in D_2_O. Note the different intensity scales. (C) Model of the proposed conformations of dimanno-AuNPs and PEG AuNPs. PEG AuNPs (left) show little order in the PEG chains. Dimanno-AuNPs (right) show a stretched-out geometry provided by the dimannose residues (organic layers not drawn to scale). The circles represent the gold core, the curved lines represent the PEG ligand, and the rigid thin line represents the PEGylated ligands ordered by the presence of dimannose residues (two hexagons), surrounded by water (droplet-like grey areas).

From symmetry rules and the ssp-polarization, one can deduce that the CH_2_ groups are again oriented, but differently from those in the PEG AuNPs. This shows directly that the dimannoside strongly affects the conformations of PEG and alkyl chains. We can exclude disorder and propose a stretched-out geometry based on the relatively narrow bands. The dimannoside must be covered by water, and, indeed, a broad peak centered around 3400 cm^−1^, characteristic of liquid water, is present [26]. Water on the dimannoside can adsorb in a multitude of orientations. This averaging results in a low signal. Figure 5 provides a sketch of the principal ideas.

VSFG experiments were repeated following exposure to D_2_O. The rationale for using D_2_O was to differentiate the hydroxy groups of sugar molecules from those associated with adsorbed or absorbed water, based on the known disparity in H–D exchange rates between sugar-bound OH groups and those in pure bulk water [29–30]. In this experiment, 30 min of exposure to D_2_O were insufficient to fully exchange the hydrogen atoms in the dimannose hydroxy groups [31]. However, given the large excess of D_2_O, it is expected to displace the thin layers of adsorbed water.

As shown in Figure 5B, this expectation is confirmed: The CH region remains virtually unchanged, while the signals near 3400 cm^−1^, characteristic of liquid water, are nearly absent. It is important to note that the OD stretch of liquid D_2_O lies outside the probing frequency window. Interestingly, under the experimental conditions, H_2_O molecules in the topmost water layer appear capable of exchanging back, as evidenced by a nonzero, tilted background-like feature between 3300 and 3700 cm^−1^. This observation supports the model of thin adsorbed water layers and further highlights the pronounced hydrophilicity of both PEG-AuNPs and dimanno-AuNPs.

### Adsorption of the particles on hydrophilic and hydrophobic surfaces

**Figure 6:**
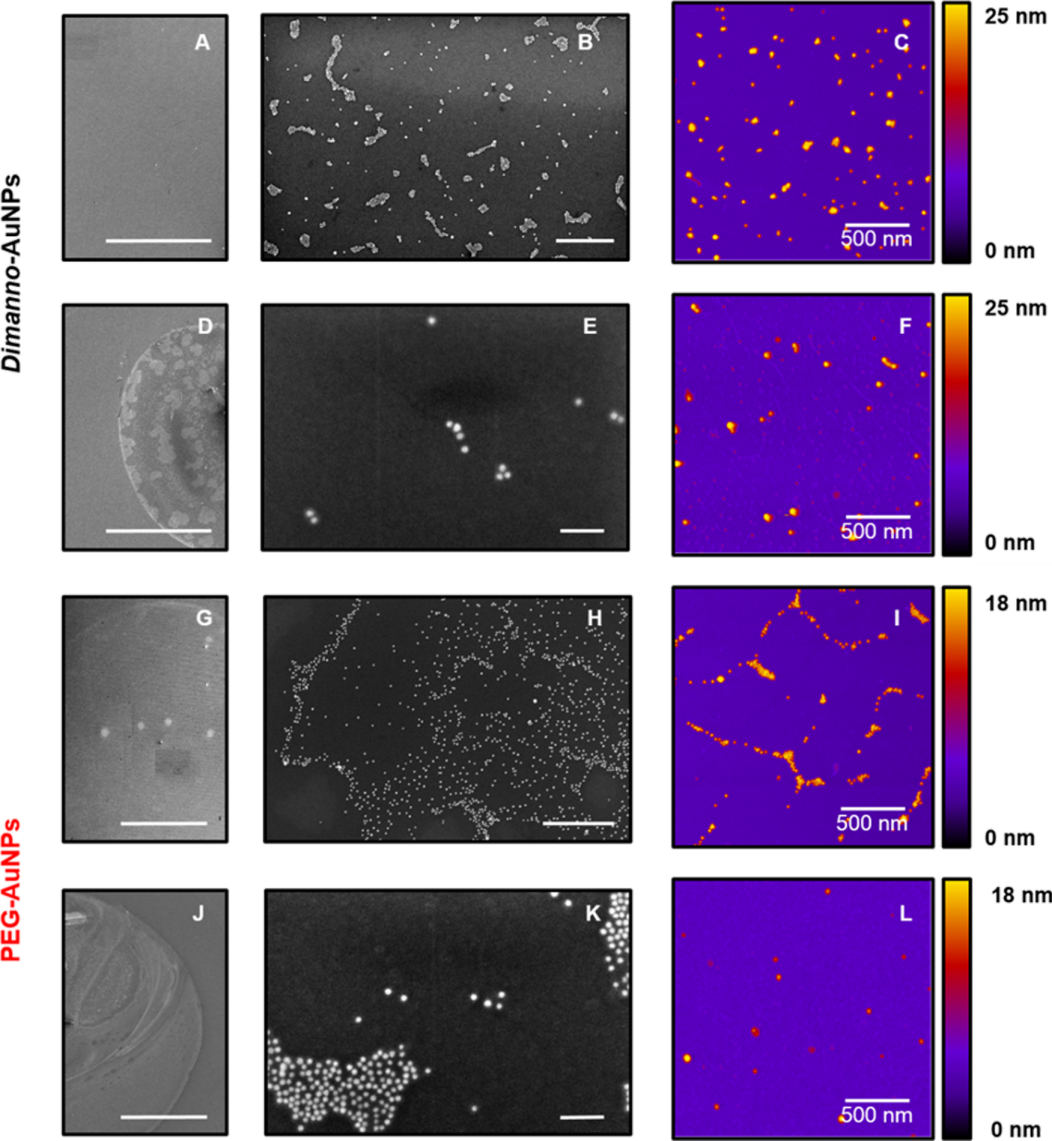
Representative SEM and AFM images of dimanno-AuNPs and PEG AuNPs on hydrophilic and hydrophobic surfaces. (A, B, D, E, G, H, J, and K) SEM images. (C, F, I, and L) AFM images. (A, C) Dimanno-AuNPs adsorbed on hydrophilic APDMES-modified wafer. (D, F) Dimanno-AuNPs adsorbed on hydrophobic OTS-modified wafer. (G, I) PEG AuNPs adsorbed on the hydrophilic surface. (J, L). PEG AuNPs adsorbed on the hydrophobic surface. Scale bars: 400 µm (A, D, G, and J), 500 nm (B, H), 100 nm (E, K). The AFM height scale is adjusted to each particle diameter.

We assessed with SEM and AFM the aggregation tendency of the particles after adsorption. To this end, we determined water contact angles. The hydrophilic APDMES silicon gave 66 ± 3º. In contrast, on the hydrophobic octadecyltrichlorosilane (OTS) silicon, it was 92 ± 2º. Comparison of the two surfaces shows characteristic differences (Figure 6), as known from analogous investigations with tobacco mosaic virus [32] and with surface-layered proteins [24].

The dimanno-AuNPs are homogenously distributed on APDMES. Consequently, SEM images have low contrast (Figure 6A). Zooming to the nanoscale, we found a homogenous dispersion of the particles (Figure 6B). This is supported by AFM topography images at the nanoscale (Figure 6C). In contrast, PEG AuNPs are not well dispersed, with more agglomerates (see the analogous Figure 6G–I), and particle clusters form microscale islands.

We analyzed adsorption on the hydrophobic OTS with the same procedure (Figure 6D–F and Figure 6J–L). Here, the required drop-casting method resulted in a typical “coffee ring” effect [33], as nicely seen in Figure 6D,J. Local inhomogeneities prevail on areas inside and outside the ring. Generally, we observed a very small number of NPs and higher agglomeration than on APDMES (Figure 6E,F,K,L). Like on APDMES, PEG AuNPs are less uniformly distributed than dimanno-AuNPs. The differences are likely due to the delicate balance between capillary flow and surface interactions. The flow is likely the dominant driver of particle motion, and strong particle–substrate interactions may lead to particle attachment before they are transported to the contact line, thereby preventing the formation of a “coffee ring” [34].

### Detailed height measurements of adsorbed particles in water vapor

AFM under variable RH was carried out under three distinct hydration conditions, which we define as (1) “low humidity”, 15 ± 5% RH, corresponding to desert climate (or very low temperatures), (2) “medium humidity”, 50 ± 5% RH, which is slightly above the standard comfort value, and (3) “high humidity”, which required overnight sample incubation to reach 90 ± 5% RH, corresponding to very wet climate or air during precipitation.

In addition, we refer to “hydrophilic conditions” in AFM for hydrophilic surfaces (APDMES silicon) scanned by a silicon tip. We found a vertical resolution (∆*z*) of 0.04 nm and a surface roughness of 0.16 nm (for scans in the 100 to 1000 nm range). Amplitude–distance curves (see Supporting Information, Section S3) suggest working regimes controlled by long-range attractive forces, both at low and high humidity. Under these conditions, we minimize sample damage by working in the attractive/non-contact regime (our amplitude curves in Supporting Information File 1, Figure S3 compare well with those reported by Garcia and San Paulo [35]). Capillary forces are present, but it is likely that the tip does not enter the water layer.

**Figure 7:**
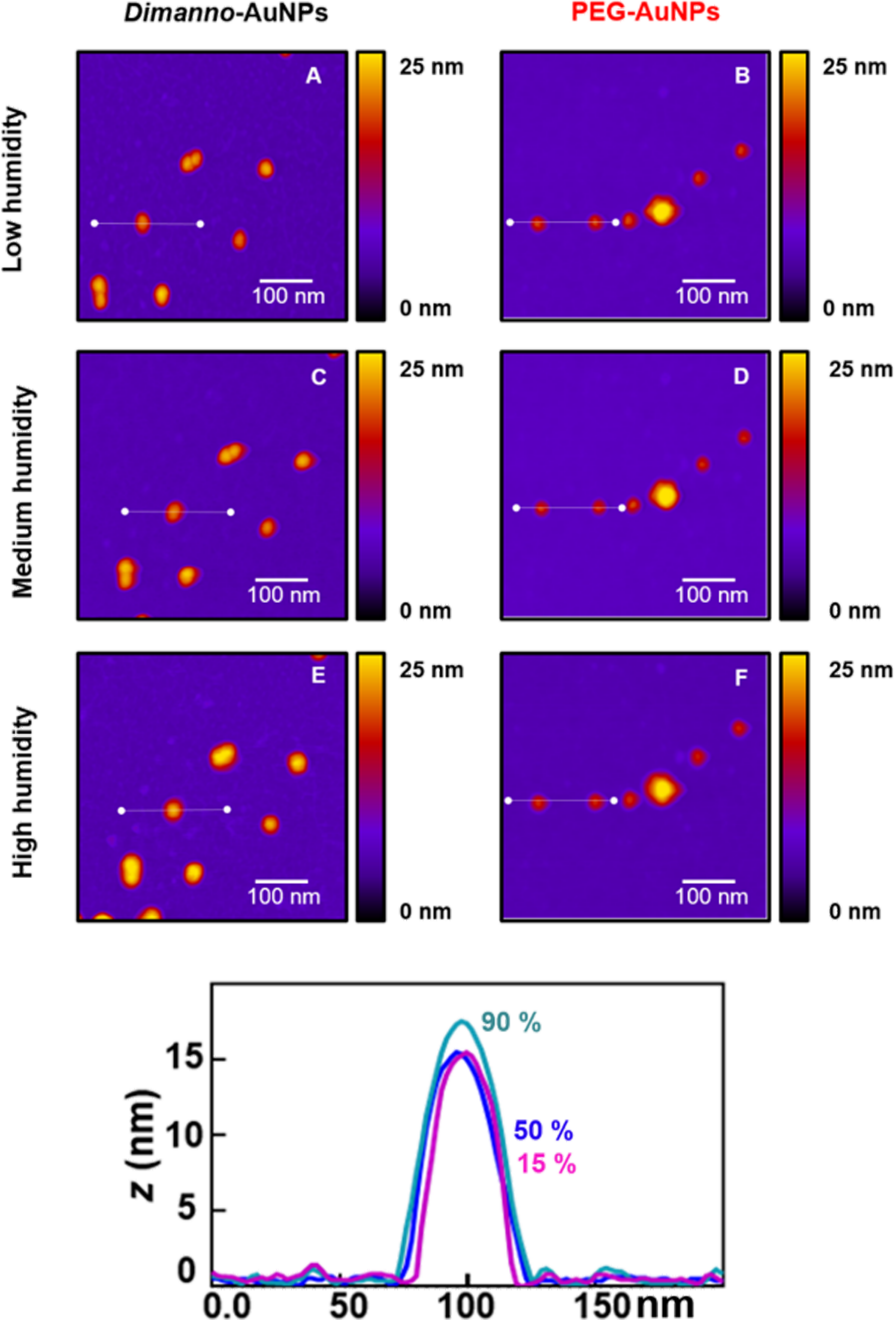
AFM topography of dimanno-AuNPs and PEG AuNPs under hydrophilic conditions (silicon AFM tip, APDMES silicon surface). (A, B) Low humidity (~15% RH), (C, D) medium humidity (~50% RH), and (E, F) high humidity (~95% RH). (A, C, E) Dimanno-AuNPs; (B, D, F) PEG AuNPs. Lower panel: Overlay of the three profiles shown in (A,C,E). Analogous data for the PEG-AuNPs are reported in Supporting Information File 1.

We imaged randomly selected NPs under the three humidity conditions and analyzed their height profiles. In the following, we focus on selected single particles. For the dimanno-AuNPs, the maximum height, *z*_max_, of the NPs in Figure 7A was 15.6 nm at ~15% RH). We found 15.8 nm, hence no measurable increase, at ~50% RH (Figure 7C), and 17.7 nm at ~95% RH (Figure 7E), a 2.1 nm increase. For PEG AuNPs, we chose two particles of 13.3 nm (left) and 12.9 nm (right) (Figure 7B). Here, *z*_max_ decreased upon humidity increment, to 12.4 nm and 11.9 nm (Figure 7D), respectively, and finally at high humidity to 12.0 nm and 11.9 nm, respectively (Figure 7F); Supporting Information File 1, Figure S4 provides additional details.

**Figure 8:**
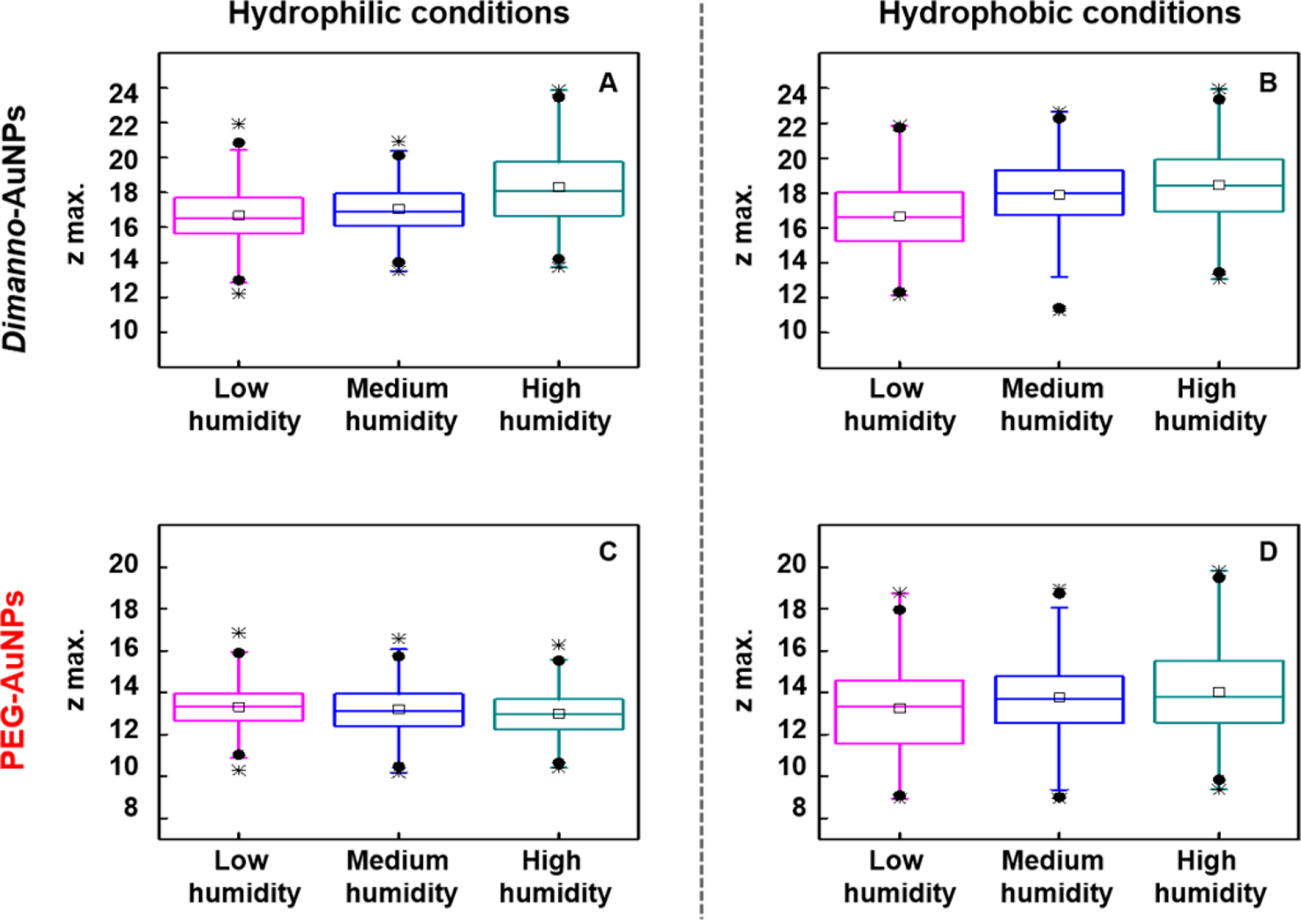
Box plots of AFM heights of dimanno-AuNPs and PEG AuNPs obtained at different humidity levels. AFM data of dimanno-AuNPs under (A) hydrophilic conditions (APDMES surface, silicon tip, ca. 500 experiments) and (B) hydrophobic conditions (OTS surface, carbon tip, ca. 250 experiments), and of PEG AuNPs under (C) hydrophilic and (D) hydrophobic conditions. Magenta boxes refer to low humidity, blue boxes to medium humidity, and dark cyan boxes to high humidity. Each box plot shows the interquartile range (from 25% to 75%). The extreme of the box represents the 5% and 95% quartile, respectively. Middle lines represent the median and the squares of the mean value. Black circles are the 1% and 99% quartile, and black stars are the minimum and maximum value.

To increase the statistical accuracy of the experiment, we tested ca. 500 randomly selected particles under the same humidity conditions (Figure 8A,C and Table 1). The resulting box plots for the hydrophilic conditions correlate well with the analysis on the selected single particles (Figure 7, see also related scans in Supporting Information File 1, Figure S4). The particle height of 16.7 ± 1.6 nm increases by 0.3 nm under medium and by 1.6 nm under high humidity. The PEG AuNPs of 13.3 ± 1.0 nm show an apparent slight reduction by 0.3 nm, but only at high humidity, which is within the uncertainty of the experiments (the errors are at least 1.0 nm, even though we measured ca. 500 NPs for each humidity). Hence, our interpretation is that we cannot detect an effect.

**Table 1:** Maximum heights (*z*_max_) of individual dimanno- and PEG-AuNPs measured by AFM under different hydration conditions. Results are expressed as the mean ± SD. Uncertainties were obtained from statistical errors. The number *n* refers to the evaluated AFM scans. We tested the reliability of our *z*_max_ values with a nonparametric Mann–Whitney test (Supporting Information File 1, Figure S6). For the dimanno-AuNPs, under hydrophilic and hydrophobic conditions, and for experiments with deuterated water, we found no significant differences in sample distribution at both low (*p* = 0.46) and high humidity (*p* = 0.06). Differences among PEG AuNPs depend on the scanning conditions, but only when comparing hydrophilic and hydrophobic conditions at high humidity (*p* < 0.0001).

Particle	Surface	Water vapor	*z*_max_ at low humidity (nm)	*z*_max_ at medium humidity (nm)	*z*_max_ at high humidity (nm)

dimanno-AuNPs	APDMES/Si, hydrophilic	H_2_O	16.7 ± 1.6 (*n* = 594)	17.0 ± 1.4 (*n* = 532)	18.3 ± 2.1 (*n* = 492)
dimanno-AuNPs	OTS/Si, hydrophobic	H_2_O	16.7 ± 2.0 (*n* = 594)	17.7 ± 2.3 (*n* = 532)	18.4 ± 2.2 (*n* = 492)
dimanno-AuNPs	gold, hydrophilic	D_2_O	16.6 ± 1.6 (*n* = 300)	17.2 ± 1.5 (*n* = 300)	17.9 ± 1.9 (*n* = 300)
PEG-AuNPs	APDMES/Si, hydrophilic	H_2_O	13.3 ± 1.0 (*n* = 505)	13.2 ± 1.1 (*n* = 495)	13.0 ± 1.0 (*n* = 499)
PEG-AuNPs	OTS/Si, hydrophobic	H_2_O	13.2 ± 2.1 (*n* = 505)	13.7 ± 2.0 (*n* = 495)	14.0 ± 2.2 (*n* = 499)

To prove that the increase in *z*_max_ is solely due to the adsorbed water, we repeated the experiments under hydrophobic conditions: We employed OTS silicon surfaces and hydrophobic carbon AFM tips. From this, we exclude that the observed phenomena is specific for the type of surface [36] or based on mechanical contact between tip and surface [22–23,37]. The ∆*z* value under ambient conditions was 0.03 nm, and the roughness of the OTS on silicon was 0.55 nm for scans between 100 and 1000 nm. In this case, it was not possible to follow the same particles along the different humidity steps due to the instability of the measurement, which did not allow for locating the scanned area after changing the humidity level. We recorded ca. 250 measurements of single particles on random positions (Figure 8B,D and Table 1). The data at variable humidity for dimanno-AuNPs fit well with the observations under hydrophilic conditions; we found an increase in *z*_max_ of 1.7 nm from low to high humidity (Figure 8). In contrast, the results of the PEG AuNPs under hydrophobic conditions differed slightly from those under hydrophilic conditions, that is, we found an increase of 0.8 nm (Figure 8).

In addition, in AFM experiments, we used air humidified with D_2_O instead of H_2_O again on dimanno-AuNPs adsorbed on gold and scanned with a silicon tip (Supporting Information File 1, Section S5). Although we expected no difference, this test was required to compare the conditions to those used in the VSFG experiments. Working with D_2_O enables exploring molecular hydrate structures [31]. Here, we aimed to identify if the increase in the maximum height of single particles was merely driven by the adsorption of water layers on top of the dimannose residues or by other mechanisms such as swelling, that is, the incorporation of water into the NP shell. As in the previously introduced cases, the particles increased their maximum height when the humidity of the system increased. This was influenced neither by the surface nor by the tip. So, our D_2_O data compares well with those under hydrophilic and hydrophobic conditions (Table 1). We evaluated 300 experiments.

## Discussion

We first discuss the spectroscopic results. FTIR and VSFG confirm the chemical composition of the organic layers (Figure 1 and Figure 5). The VSFG results additionally give sensitive information on the local ordering of the molecular chains in the ligands. We conclude that the dimannose residues are coupled to the PEG chains and provide specific ordering properties: Under dry conditions, both PEG and dimannoside bind water. However, the higher hydrophilicity of dimannoside enables a better interaction with water molecules, which remain bound even after dehydration. This is also supported by research on SAMs on gold surfaces, which reveal a more hydrophilic tendency of surfaces functionalized with mannose terminal groups (C_6_H_12_O_6_C_5_S, contact angle <5°), compared with carboxylic acids (COOHC_11_S, contact angle 44 ± 1º) [38]. Whenever NPs cluster, the steric hindrance effects of the bulky dimanno-AuNPs should additionally create nanoscale “traps” between the NPs. Both effects result in water adsorption that persists even in dry environments.

Focusing now on particle size and assembly, we found no difference between the hydrodynamic size of 16.8 nm (obtained in suspension by DLS) (Figure 2) and the AFM height of 16.7 nm (which we here limit to the case of complete dehydration) (Table 1). DLS usually gives higher particle sizes due to the hydration layer [39–40], which slows the diffusion of NPs in aqueous solutions, especially our hydrophilic NPs. We suggest two possible explanations. First, the dimannose residues could be very efficiently packed (with PEG ligands acting as spacers); second, the adsorption of the NPs distorts the ligands between the NP core and the solid surface. The latter effect is well known from adsorbed NPs, where one should consider up to three ligand spaces, namely, ligands between NP and surface, ligands that stretch out parallel to the surface in contact with it, and free ligands merely attached to the NPs.

For the PEG AuNPs, DLS gave an extreme value of 41.1 nm, corresponding to three times the AFM height value (13.3 nm). We conclude that we here observe particle agglomeration in solution. Previous studies have shown such clusters for PEG AuNPs in solution [41], and their size is strongly reduced by adding albumin. In our case, the addition of dimannose would lead to the case of the dimanno-AuNPs, for which we do find much decreased agglomeration. Generally, nanoparticles produced inside a concentrated carbohydrate solution (more than 40% of the total aqueous volume) provide a macromolecular crowding environment which diminishes particle interaction [42]. The dimanno-AuNPs used in this research were produced in a solution of ca. 50% dimannoside ligand, sufficient to decrease the agglomeration tendency. The observed excellent stability of dimanno-AuNPs may be associated with the physical constraint of the dimannoside being present during synthesis and the strong intra-NP hydrogen bonding interactions.

These properties are also reflected in the macroscale structures formed upon adsorption. However, the surface is crucial here, and our hydrophilic APDMES- and hydrophobic OTS-silanized wafer surfaces (see Supporting Information File 1, Figure S1) provide two extremes (Figure 6). We observed a “coffee ring” drying phenomenon only on OTS. This is related to the competition between the time scales of the liquid evaporation and the particle movement: The solvent evaporates so slowly that particles move over at least mesoscale distances, thus allowing for aggregation in the ring [33]. In our research, the deposition protocol may have influenced the observed phenomena. The hydrophilic APDMES favors NP–surface interactions with subsequent immobilization. This can be based on multiple hydrogen bonds between the NH_3_^+^ groups in APDMES and the OH groups in the dimannoside, which immobilize the NPs long before solvent evaporation. Consequently, in this case, we observed no “coffee ring” effect. The drop-casting method used on OTS allowed for complete evaporation of the droplet. The NP–surface interactions are now relatively weak, and inter-NP interactions are favored. NP movement was faster than evaporation, resulting in the observed “coffee ring” drying.

Mesoscale observations of NP assembly by AFM and SEM complement the results discussed above. The use of hydrophilic and hydrophobic surfaces and also AFM tips allows one to distinguish ubiquitous water layers, present on all surfaces, from specific water adsorption on the AuNPs. For the APDMES surface at neutral pH, we expect electrostatic interactions between the NH_3_^+^ groups and the carboxylate residues on the NPs, and hydrogen bonds. Hence, there are relatively few differences between dimanno-AuNPs and PEG AuNPs, which are related to the particle dispersion in solution. The clustered PEG AuNPs cannot disperse well on a surface, while the single dimanno-AuNPs interact efficiently, indicating that NP–surface interactions dominate in this case over NP–NP interactions. We ascribe this to hydrogen bonds between the (multiple) OH groups in the dimannoside and the amine. This finding compares well with TEM on carbon grids: PEG AuNPs with short or long ethylene glycol chains adsorb in clusters [43], while glycosylated AuNPs are homogeneously dispersed [10].

Our AFM observations of nanoscale hydration [44] show a general trend to stronger water adsorption on dimanno-AuNPs, as compared to the PEG AuNPs (Figure 7). This agrees with AFM hydration studies of sucrose particles, which become liquid above 60% RH [45]; hence, the more hydrophilic nature of the dimannose residues is expected. Statistical analysis demonstrates that only the water adsorption on dimanno-AuNPs is independent from the scanning conditions (Table 1 and Supporting Information File 1, Figure S6). Between hydrophilic and hydrophobic conditions, we find only small differences, which we interpret as quasi-identical ubiquitous water layers on our silanes. Although APDMES would be expected to bind more water than OTS, this increase is smaller than our experimental error.

Considering the size of a water molecule (~0.28 nm) and the typical thickness of hydration layers (~0.6 nm) [21–22,44,46–47], the absolute values of water adsorption on dimanno-AuNPs fit to three to four layers of water (~1.5 nm). STEM found that the organic layer on dimanno-AuNPs is ~1.5 nm (Figure 4). Hence, we propose a 100% thickness increase due to water adsorption or absorption, that is, the water can be adsorbed in a layer or absorbed among the various dimannoside groups. The latter case would correspond to a “swelling” of the AuNP coating, which is agreement with microbalance observations of highly hydrated cellulose films [48], which found 3.6 mol water per cellulose subunit at 97% RH.

Under hydrophobic conditions, PEG AuNPs show a moderate increase in AFM height, corresponding to one water layer, not observed under hydrophilic conditions. It is possible that this is based on reproducibility issues, which are well known for hydrophobic samples. These can be based on irreversible modification of tip or sample, or even both [37]. In this regard, wet STEM and SEM investigations on PEG AuNPs differ from our data, indicating that particle hydration is independent of the selected surface [21–22,36,49]. However, these studies were performed on particle clusters in crowded environments, which induce collective phenomena [36,49] that are not in our scope.

## Conclusion

Gold nanoparticles (AuNPs) have been widely investigated for biomedical applications like biochemical sensing, imaging, or drug delivery systems. The success of these platforms stems from their dispersion in water, stability, and biocompatibility in fully hydrated states, as well as in biological fluids. Our investigation shows a novel approach to these particles by testing the hydration properties under different air humidity conditions. We characterized AuNPs coated by oligo(ethylene glycol) (PEG) and dimannoside with a multimethod approach (VSFG, FTIR, DLS, ZP, SEM, STEM, and AFM). We proved that various properties, mainly those of the adsorbed dimanno-AuNPs, depend on environmental conditions, specifically, humidity and the hydrophilicity of the adsorption surface.

Our spectroscopic investigations verified the known chemical properties. With VSFG under controlled humidity, we found ordering phenomena in ligand chains, which we attribute to forces exerted by hydrated dimannoside. Such an ordering is absent in conventional PEG ligands. Our observations of nanoscale hydration by AFM in a humidity chamber show a general trend of stronger water adsorption on dimanno-AuNPs than on the PEG AuNPs. Statistical analysis and detailed tests of hydrophilic and hydrophobic surfaces, with both hydrophilic and hydrophobic AFM tips, helped us to exclude the role of water adsorbed on the surfaces. We found a 100% thickness increase of the 1.5 nm thick dimannoside ligand shell (i.e., 1.5 nm of water), corresponding to about four layers of water. The water can be absorbed in a layer or absorbed among the various dimannose groups.

The increased water adsorption of dimanno-AuNPs, compared to the well-studied PEG AuNPs, makes them candidates for gas sensing applications. Sensors are usually kept dry and then become hydrated in standard environments. Whenever dry conditions are problematic, our study suggests testing carbohydrate coatings.

Finally, revealing the water adsorption on dimannoside from dryness to high humidity offers new insights into the molecular role of these surface residues also from a biophysical perspective, which is especially valuable when the biological objects in question are highly pathogenic. Size, surface composition, and controlled orientation of the dimanno-AuNPs suggest suitable candidates for emulating viral surface glycoproteins, such as influenza hemagglutinin, under hydration–dehydration cycles in air, which correspond to the conditions of viral transmission. Indeed, several viral pathogens display mannose residues on their surface, particularly surface glycoproteins. These are responsible for infection, but might also provide adaptability to harsh environments, such as dry conditions. Our approach is yet focused exclusively on water; future investigations should include the effects of adsorbed salts and proteins, and the temperature.

## Experimental

### Materials

Acetone (99.5%), 3-(ethoxydimethylsilyl)propylamine (APDMES, 97%), and anhydrous solutions of toluene, chloroform, octadecyltrichlorosilane (OTS), and decalin (*cis* and *trans*) were purchased from Sigma Aldrich (Germany). 2-Propanol (99.5%) was obtained from Acros (Belgium) and absolute ethanol (99%) from Panreac (Spain). Aqueous solutions of dimanno-AuNPs and PEG AuNPs were kindly provided by the Bio Nano Plasmonics Lab at CIC Biomagune (San Sebastián, Spain). Water was of ultrapure quality (Milli-Q, 18.2 MΩ·cm, <10 ppb total organic content).

### Synthesis of gold nanoparticles

Gold nanoparticles were synthesized by the Bio Nano Plasmonics Lab at CIC biomaGUNE according to [10]. Both PEG AuNPs and dimanno-AuNPs were obtained by reduction of AuCl_4_^−^ with BH_4_^−^ in the presence of the corresponding thiol ligands. For the PEG AuNPs, a PEG thiol with carboxylic acid termination was used. To obtain the dimanno-AuNPs, 50 mol % of the same carboxylic acid ligand was mixed with 50 mol % of a dimannoside (Manα1-2Man) thiourea PEG thiol. After purification and lyophilization, the nanoparticles were obtained as brown powders.

### Dynamic light scattering and zeta potential measurements

DLS was used to determine the hydrodynamic diameter, and ZP was used to estimate the NP surface charge of both particles in solution. We employed Zetasizer Nano ZS (Malvern Panalytical, UK) equipment. For DLS measurements, 70 µL of the sample (1.0 × 10^12^ particles/mL) was loaded in a previously Milli Q-washed microcuvette at 25 °C and a detection angle of 173°. For ZP, 700 µL of the sample was loaded in a dip-cell cuvette. Three runs were performed in three replicates, resulting in nine measurements per sample. The hydrodynamic diameter (z-average) and the NP surface charge (ZP) were calculated with the equipment software (v.7.12) without any further data processing, hence assuming spherical shapes. For ZP, we set the factor *f*(*K*α) = 1.5 and used the Smoluchowski approximation.

### Preparation of hydrophilic and hydrophobic surfaces

Dimanno-AuNPs and PEG AuNPs were adsorbed on silicon wafers, which we functionalized with APDMES or with OTS (Supporting Information File 1, Figure S1), referred to as “hydrophilic” or “hydrophobic”, respectively. The silicon wafers were initially chemically cleaned by successive serial sonication steps of 5 min each using acetone, 2-propanol, and absolute ethanol and then dried with a nitrogen stream. Oxygen plasma etching (Diener, DE) was carried out for 8 min with 300 W of nominal power (100%), at millibars of oxygen pressure, which creates an OH-terminated layer of hydrophilic silicon oxide.

For the hydrophilic functionalization, the surfaces were subsequently immersed in 2 mL of APDMES in toluene (1:100 v/v) and heated to 60 °C for 30 min. To avoid the hydrolyzation of APDMES, the procedure was achieved in a glove box. After functionalization, the surfaces were rinsed in toluene and dried in flowing nitrogen.

The hydrophobic functionalization was performed as reported in [24]. Silicon wafers were washed first in methanol, then in a 1:1 v/v mixture of methanol and chloroform, and finally in chloroform under 5 min sonication at every step. Afterwards, the surfaces were immersed in a 7:2:1 (v/v) mixture of decalin (cis and trans)/toluene/chloroform, and 0.1% OTS was added. The procedure was done at ambient temperature inside a nitrogen glove box. After 12 h of incubation, the surfaces were rinsed with chloroform, then with a 1:1 (v/v) mixture of chloroform and methanol, and finally with methanol. The process was finished by drying the surfaces with a stream of nitrogen.

### Contact angle measurements

For static contact angle measurements, sessile drop experiments were performed in triplicates at ambient temperature (23–25 °C) with a standard contact-angle measurement system (G10 goniometer, Krüss). A droplet of ultrapure water of 4 µL volume was placed onto the functionalized silicon surfaces and contact angles from the drop profile were measured.

### Adsorption of nanoparticles

Dimanno-AuNPs or PEG AuNPs solutions were incubated for 5 min (1.0 × 10^12^ particles/mL) on the hydrophilically functionalized silicon surfaces. The samples were washed with Milli-Q water and dried in flowing nitrogen. The adsorption on the hydrophobic surfaces was achieved by drop casting. A droplet of dimanno-AuNPs or PEG AuNPs was deposited on the surface and dried in air.

### Infrared spectroscopy

FTIR measurements were carried out with a grazing incidence objective lens/mirror system in a Hyperion 2000 microscope with a Vertex 70 spectrometer (Bruker). For this, a concentrated solution of particles (1.9 × 10^13^ particles/mL) was cast on a carefully cleaned gold surface and evaporated under ambient conditions. The measurements were achieved by recording 2000 scans, from 650 to 4000 cm^−1^ at a resolution of 4 cm^−1^, in triplicate. Background spectra were recorded on gold areas outside the evaporated droplet. The FTIR spectra were obtained with the equipment software OPUS v.6.5 without further data processing.

### Sum frequency generation spectroscopy

VSFG spectra of dimanno-AuNPs and PEG AuNPs was recorded in a broad-band VSFG system at Fritz-Haber-Institute (Max-Planck-Gesellschaft, Berlin, Germany). Gold thin films (200 nm on 10 nm Cr on glass) were used as surfaces. Prior to sample deposition, the surfaces were cleaned with ethanol and Milli-Q water under sonication for 10 min. UV/ozone cleaning was applied to remove any possible hydrocarbon contamination, and the VSFG spectrum of washed gold was used as reference to normalize the non-flat infrared power distribution, similar to FTIR recording. About 10 µL of the sample was drop-cast on the surface and evaporated under nitrogen flow. The VSFG experiments were achieved under dry air flushing at room temperature (24 °C) at four different center frequencies to measure the complete CH and OH stretching region in the broadband infrared. The tunable infrared laser produced femtosecond pulses of ca. 5.5 mW (at 3300 cm^−1^). The focal length was 30 cm with an incident angle of 45°. The visible picosecond pulses were at a fixed frequency of 800 nm (12500 cm^−1^) and of 10 mW power. The focal length was 100 cm with an incident angle of 65°. The sum frequency signal was collected in reflection, accumulated for 1 min for ppp polarization (all beams polarized normal to the surface) and 2 min for ssp (laser beams polarized parallel to the surface, sum frequency emission and up-conversion polarized perpendicular and infrared parallel to the plane of incidence).

### Electron microscopy imaging

SEM and STEM images were obtained in a Helios NanoLab 450S dual beam microscope (FEI NL/Thermo Fisher Scientific). SEM images were obtained of dimanno-AuNPs and PEG AuNPs at 5 kV with 50 pA current in high-vacuum mode. The core diameter of single particles was manually counted with ImageJ (NIH, https://imagej.nih.gov/ij/). The number of particles was expressed as *n*, and the data were displayed as the mean ± SD. Totals of 378 particles for the dimanno-AuNPs and of 491 for the PEG AuNPs were counted.

STEM acquisition was achieved in high vacuum at 30 keV and 50 pA current on negatively stained dimanno-AuNPs samples. For the negative staining, 0.35 µL of dimanno-AuNPs (1.9 × 10^13^ particles/mL) were deposited on glow-discharged carbon TEM grids (air flow, 20 mA, 4 min). Then, 0.35 µL uranyl acetate (0.5% *w*/*w*) was deposited and dried on the sample.

### AFM imaging

AFM topography images were recorded with an Agilent 5500 AFM (Keysight, Scientec, FR), in air in AC “noncontact” mode at 24 °C, at speeds between 0.8 and 1.2 lines/s. The scanning was performed in at least six replicates per condition for the PEG AuNPs and nine replicates for the dimanno-AuNPs.

Silicon cantilevers (Multi 75, Budget Sensors) with a force constant *k* = 3 N/m, a resonance frequency *f* = 75 kHz, and a nominal radius of less than 10 nm were used for the experiments on the hydrophilic surfaces. These conditions are referred to as “hydrophilic conditions”.

The visualization of particles on hydrophobic surfaces was achieved by employing gold-coated silicon probes with a 1 nm carbon spike (*k* = 5 N/m, *f* = 160 kHz, Hi’Res C14/Cr Au, MikroMasch). These conditions are referred to as “hydrophobic conditions”.

The samples were incubated on the hydrophilic or hydrophobic surfaces, and AFM topography images were taken under humidity-controlled atmospheres at 15 ± 5%, 50 ± 5%, and 90 ± 5% RH. These humidity conditions are referred to as “low humidity”, “medium humidity”, and “high humidity”, respectively. For this, an environmental isolation chamber (Keysight) was mounted to the AFM, thus providing a completely sealed and isolated environment. The RH of 15 ± 5% was achieved by pumping nitrogen to the isolation chamber. The RH of 50 ± 5% was reached by controlling the mixing ratio between a dry and a wet stream of nitrogen, produced in a bubble flask. To reach a RH of 90 ± 5%, the sample was allowed to equilibrate overnight in the saturated chamber. The images were examined by using picoView 1.14 software (Keysight). AFM images were analyzed after scanning with WSxM 5.0 Develop software version 8.0 [50] for obtaining the maximum height of the particles and height profiles. For this, the images were processed by levelling the plane of the image and applying parabolic flattening. Around 500 individual particles were processed for the hydrophilic conditions and 250 for the hydrophobic conditions. The particles were manually selected by using the criterion of single-particle observation with FWHM values below 30 and 40 nm for the hydrophilic and hydrophobic conditions, respectively. Gwyddion software version 2.47 was used for displaying the topography images. The images were first levelled by mean plane subtraction and aligned by height median; occasionally, the *z* excursion outliers were manually removed by, for example, correction of the scan line artefacts in the *x*-axis or misaligned segments within a single row. The height distribution histograms were calculated with Origin 8.0 software, and height profiles were obtained with GraphPad Prism version 8.0.1. The number of particles was expressed as *n*, and the data were displayed as the mean ± SD.

### Statistical analysis

Statistical analysis was performed using GraphPad Prism version 8.0.1. Data sets were analyzed using unpaired *t*-tests (Mann–Whitney test) to compare identical particles on different surfaces at the same humidity. Two-tailed P values were reported in all the cases, and the alpha level was kept at 0.05.

## Supporting Information

File 1 Additional experimental information and spectra.

## Data Availability

Data generated and analyzed during this study is available from the corresponding author upon reasonable request.
