# Supplementary material for: Ultrathin water layers on mannosylated gold nanoparticles
Source: Beilstein J Nanotechnol. 2025 Dec 4;16:2183–98. doi: 10.3762/bjnano.16.151 (PMC12684429; doi:10.3762/bjnano.16.151)
Supplement: File 1 — Additional experimental information and spectra. [file Beilstein_J_Nanotechnol-16-2183-s001.pdf]

BEILSTEIN JOURNAL OF NANOTECHNOLOGY

Supporting Information

for

Ultrathin water layers on mannosylated gold nanoparticles

Maiara A. Iriarte Alonso, Jorge H. Melillo, Silvina Cerveny, Yujin Tong
and Alexander M. Bittner

Beilstein J. Nanotechnol. **2025,** *16,* 2183–2198. doi:10.3762/bjnano.16.151

Additional experimental information and spectra

License and Terms: This is a supporting information file under the terms of the Creative Commons Attribution License (https://creativecommons.org/licenses/by/4.0). Please note that the reuse, redistribution and reproduction in particular requires that the author(s) and source are credited and that individual graphics may be subject to special legal provisions.
The license is subject to the *Beilstein Journal of Nanotechnology* terms and conditions: (https://www.beilstein-journals.org/bjnano/terms)

S1 Hydrophobic and hydrophilic surfaces by silanization of oxidized silicon wafers

The silanes shown in Figure S1 were employed (see main manuscript text for the procedure) to produce hydrophilic and hydrophobic flat surfaces, respectively.

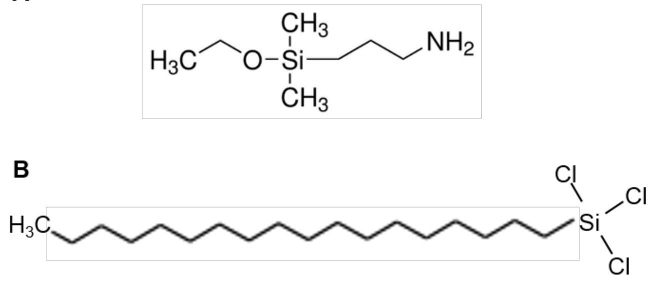

Figure S1: Molecular structures of silanes. (A) 3-(Ethoxydimethylsilyl)propylamine (APDMES) and (B) *n*-octadecyltrichlorosilane (OTS).

S2 FTIR spectroscopy

We recorded FTIR spectra in dry nitrogen; hence, the samples were at humidities well below 5%. Figure S2 shows that the PEG AuNPs show the characteristic absorption bands of PEG, whereas the dimanno-AuNPs show a very different spectrum. In either case, however, it is obvious that an organic shell is present on the AuNPs. To assign the IR bands, we used experimental spectra of water, mono-, di-, and triethylene glycol, as well as of methoxydiethylene glycol, arabinose, glucose [1], and of poly(ethlyene oxide), triethylene glycol dimethyl ether, didecylsulfide, diethylsulfide, decane, ethoxyacetic acid, acetic acid, silver, sodium, and potassium acetate, diethylthiourea, and L-mannose [2]. We furthermore employed simple simulations (based on semiempirical quantum mechanics) of gas-phase IR spectra of water and some of our ligands (dimannose, triethylene glycol, $HO(CH_2CH_2O)_3CH_2COOH$, $HS(CH_2)_{10}(OCH_2CH_2)_2OH$, and $HO(CH_2CH_2O)_2CH_2CH_2NHCSNHCH_2CH_2OH$) to clear up doubtful assignments [3,4]. We cross-checked the simulations with gas-phase spectra of water and triethylene glycol [5]. We also consulted [6-8].

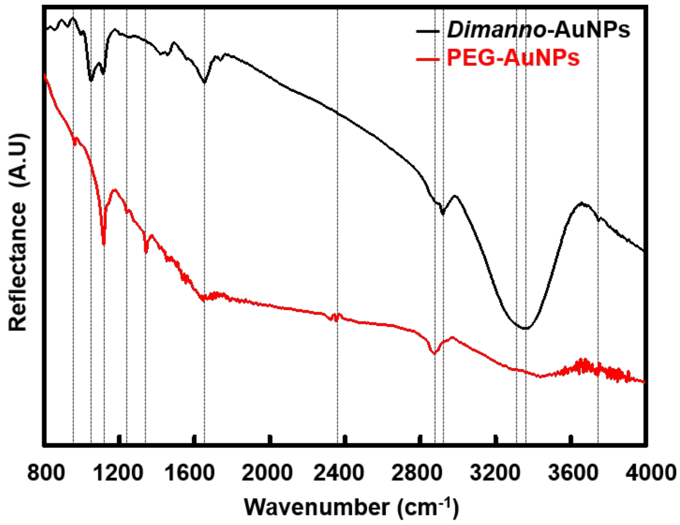

Figure S2: Grazing incidence FTIR spectra of dimanno-AuNPs and PEG AuNPs on a gold surface, normalized to pure gold, without background correction. The black line represents the infrared spectra of dimanno-AuNPs, and the red line represents the infrared spectra of PEG AuNPs. The vertical lines indicate the characteristic peaks.

PEG-AuNPs

The band at 950 cm^{-1} is well resolved. Its origin is hard to elucidate, and we propose a disordered EG chain (smaller cyclic EGs show this complex band). The strongest band at 1110 cm^{-1}, based on asymmetric C–O–C stretching, appears only as a shoulder. The signal at 1260 cm^{-1} can be assigned to the very strong C–O stretching in COOH (protonated carboxylate), although there are rather few COOH groups. The remainder of the fingerprint region is masked by H_2O (apparent as "noise" from 1350 to 2150 cm^{-1}) and CO_2 (2350 cm^{-1}) traces in the gas phase. The second strongest band at 2880 cm^{-1} is based on C–H stretching in the CH_2 groups. This band has a substructure, based on symmetric and asymmetric stretching, which is usually not resolved in EGs. There is no trace of OH groups, which are sometimes present due to unwanted hydrolysis (O–H stretching at ~3500 cm^{-1}), or from adsorbed water (H–O–H bending at 1640 cm^{-1}). These very broad bands are found in liquid water, but also in impure or not completely dried compounds. A good example is triethylene glycol dimethyl ether (1,2-bis (2-methoxyethoxy) ethane).

Dimanno-AuNPs

Although the same alkyl-PEG derivate chains must contribute, the alkyl-PEG-thiourea-dimannose shell totally dominates. This already follows from the main feature of the spectrum, a very broad and extremely intense feature of two overlapping bands at 3250 and 3400 cm^{-1}, which stems from O–H stretching vibrations (the contribution from N–H can only be from the thiourea group and must be much smaller than that from O–H). While adsorbed or absorbed water can also contribute here, this feature is assigned to the dimannoside OH groups. The band at 3700 cm^{-1} is from free (not hydrogen-bonded) OH groups. The band at 1050 cm^{-1} cannot easily be assigned (it contains C–O stretchings), but regularly occurs in sugars, while the 1120 cm^{-1} band is again from C-O-C stretching. This low-energy fingerprint region is thus based on PEG and alkyl chains, present everywhere on the NP surface. It is furthermore noteworthy that the thiourea group does not result in any strong bands (expected at 1500 and 1550 cm^{-1}, as deduced from dialkyl thiourea spectra and simulations). Water is responsible for the 1650 cm^{-1} band, assigned to H–O–H bending from adsorbed or absorbed molecules. Because we can exclude this on the pure alkyl PEG shell (see above), we assign these water molecules to the dimannose residues. Indeed, sugars are very difficult to dry completely, such that this band is easily found in many carbohydrates. The spectrum shows again a band from CO_2 in the gas phase (2350 cm^{-1}). The C–H stretching region is rather different from that of the PEG-AuNPs, though, with two bands at 2850 cm^{-1} (symmetric C–H) and 2920 cm^{-1} (asymmetric C–H). These positions are typical for alkyl chains but are also found in carbohydrates. The main differences are thus found in the O–H stretching region (3000–3500 cm^{-1}), from which we can deduce a larger concentration of OH groups in dimanno-AuNPs.

S3 AFM amplitude–distance curves

We verified that the AC (“noncontact”) AFM amplitude decreases linearly from large distances (tens of nanometers, quasi zero force) to zero when the surface is hit (Figure S3). A monotonous (here even linear) decrease is important to allow for scanning the surface of liquids.

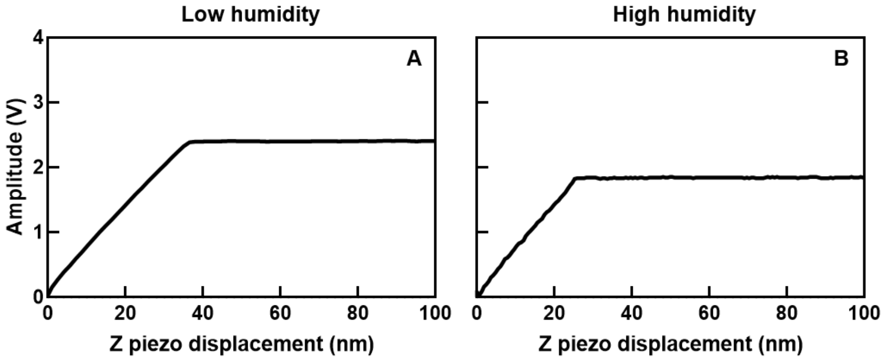

Figure S3: AFM amplitude versus *z*-piezo displacement under hydrophilic conditions, (A) obtained at low humidity (~15 % RH) (setpoint 2.2 V, drive amplitude 1.2 %), (B) obtained at high humidity (~95 % RH) (setpoint 1.9 V, drive amplitude 6 %). In both cases, the free amplitude (A_0) was 4 V.

S4 AFM scan profiles for selected particles

Figure S4 shows additional data, which follow Figure 7 (main manuscript text). At high humidity, the lateral size of both type of particles increases. This fact can depend on a non-optimized setpoint or can indicate water accumulation at the NP sides. Water condensation in the gap between particle and solid surface is indeed expected [9].

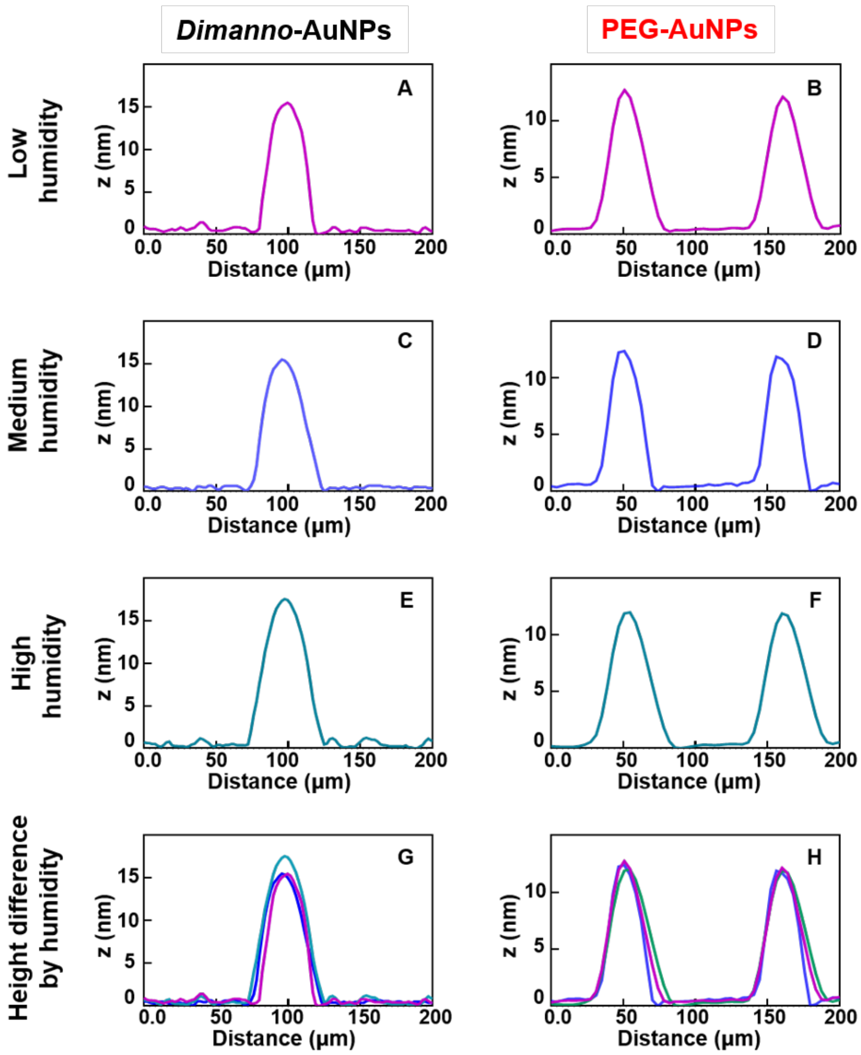

Figure S4: Representative AFM topography height profiles for Figure 7. (A, B) Height profiles of the samples at low humidity (~15% RH), extracted from the horizontal lines of (A, B) in Figure 7, respectively. (C, D) Height profiles of the samples at medium humidity (~50% RH) extracted from the horizontal lines of (C, D) in Figure 7, respectively. (E, F) Height profiles of the samples at high humidity (~95% RH) extracted from the horizontal lines of (E, F) in Figure 7, respectively. (G, H) Comparison of the scanned particles at low humidity (magenta) medium humidity (blue), and high humidity (dark cyan).

S5 AFM experiments in air humidified with D_2O

Figure S5 shows data obtained in D_2O atmosphere to verify that the system is geometrically identical to the one investigated in H_2O vapor.

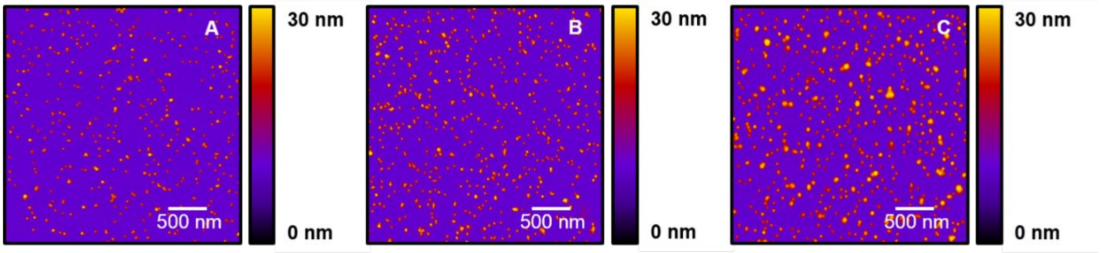

Figure S5: Representative AFM topography images of dimanno-AuNPs on gold obtained at variable humidity. (A) Particles scanned at low humidity. (B) Particles scanned at medium humidity. (C) Particles scanned at high humidity. Note that the medium and high humidities were achieved by using a stream of air/D_2O.

S6 Mann–Whitney test of z_{max}

We tested the reliability of our results with a nonparametric Mann–Whitney test, presented in Figure S6, which compares the AFM height distribution in terms of z_{max}, for low and high humidities, independently for the dimanno-AuNPs and for the PEG-AuNPs. The statistical analysis for the dimanno-AuNPs on hydrophilic and hydrophobic conditions and experiments with deuterated water show no significant differences in sample distribution at both low ($p = 0.46$) and high humidities ($p = 0.06$). In contrast, the z_{max} values obtained from PEG-AuNPs depend on the scanning conditions. Non-significant differences in height distribution are found at low humidity ($p = 0.51$), but significant differences are found when comparing the hydrophilic and hydrophobic conditions at high humidity ($p < 0.0001$).

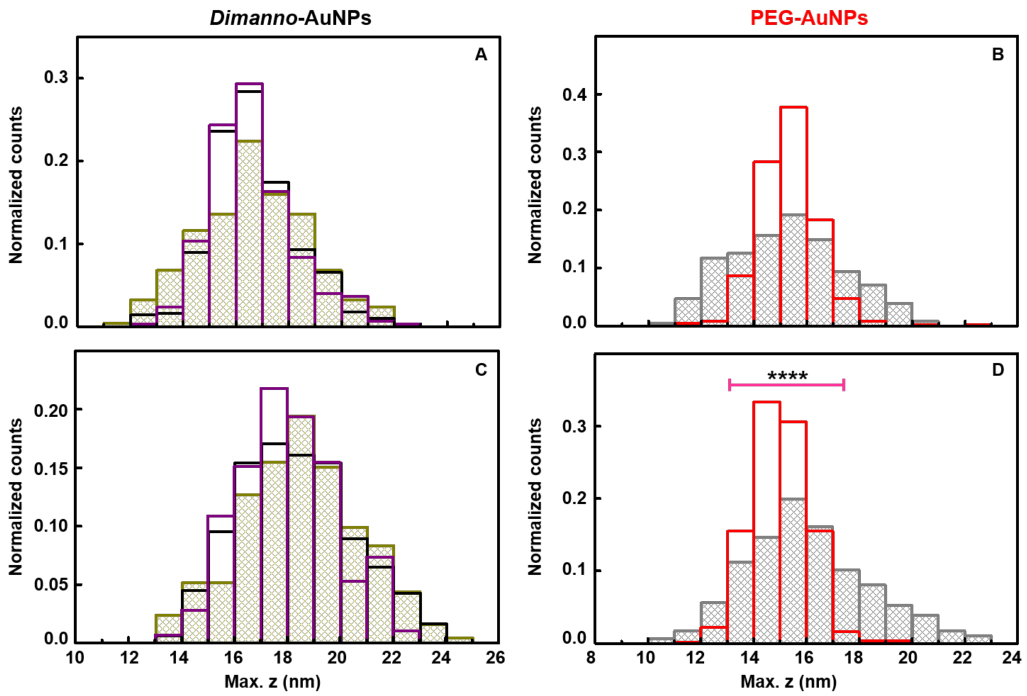

Figure S6: AFM height histograms (z_{max}) of individual dimanno-AuNPs and PEG-AuNPs achieved at low and high humidity. (A) Dimanno-AuNPs at low humidity. (B) PEG-AuNPs at low humidity. (C) Dimanno-AuNPs at high humidity. (D) PEG-AuNPs at high humidity. Yellow bars: measurements performed on gold. Purple and grey bars: measurements performed under hydrophobic conditions. Black and red bars: measurements performed under hydrophilic conditions. The pink horizontal line in (D) refers to a meaningful increase in z_{max} of the particles scanned under hydrophobic conditions with respect to the hydrophilic conditions (****$p < 0.0001$). Note that the counts are normalized.

References

1. Schrader, B. *Raman/infrared atlas of organic compounds*, 2nd ed.; VCH: Weinheim, 1989.
2. Database National Institute of Advanced Industrial Science and Technology, AIST. https://sdbs.db.aist.go.jp (accessed March 16, 2025)

3. Infrared spectra prediction. https://www.cheminfo.org/flavor/bioориented/IR/IR_spectra_prediction/index.html (accessed March 16, 2025)
4. Jablonka, K. M.; Patiny, L.; Smit, B. *J. Chem. Educ.* **2022,** *99,* 561–569. doi:10.1021/acs.jchemed.1c01101
5. NIST Chemistry WebBook. https://doi.org/10.18434/T4D303 (accessed March 16, 2025) doi:10.18434/T4D303 (accessed March 16, 2025)
6. 21.3: Spectroscopy of Carboxylic Acids. https://chem.libretexts.org/@go/page/45921 (accessed April 15, 2023)
7. Wells, H. A.; Atalla, R. H. *J. Mol. Struct.* **1990,** *224,* 385–424. doi:10.1016/0022-2860(90)87031-R
8. Lin-Vien, D.; Colthup, N. B.; Fateley, W. G.; Graselli, J. G. *The Handbook of Infrared and Raman Characteristic Frequencies of Organic Molecules;* Academic Press: San Diego, CA, USA, 1991
9. Calo, A.; Eleta-Lopez, A.; Ondarcuhu, T.; Verdaguer, A.; Bittner, A. M. *Molecules* **2021,** *26,* 5184. doi:10.3390/molecules26175184
